# Presence, flow, and narrative absorption questionnaires: a scoping review

**DOI:** 10.12688/openreseurope.13277.2

**Published:** 2021-11-04

**Authors:** Federico Pianzola

**Affiliations:** 1 Department of Human Sciences for Education "R. Massa", University of Milan Bicocca, Milan, Italy; 2 School of Media, Arts and Science, Sogang University, Seoul, South Korea

**Keywords:** Presence, flow, narrative absorption, immersion, scoping review, questionnaires

## Abstract

**Background**: This is a review and analysis of the questionnaires most used in empirical research on psychological phenomena labelled as “presence,” “flow,” and “narrative absorption,” mostly for experiences mediated by technology (printed books, screens for games and films, and virtual reality). Overlapping concepts have been formulated in different fields according to specific disciplinary interests and based on knowledge within each field.

**Objectives**: This review focuses on how language is actually used in questionnaire items, rather than on how concepts are formulated top-down and associated with corresponding linguistic expressions that become items of a questionnaire. The goal is to highlight similarities and overlaps in order to show a possible interdisciplinary agreement about the core aspects of the psychological states elicited by mediated experiences.

**Eligibility criteria**: Questionnaires developed or used for research about VR, video games, films, or books have been selected for analysis. They should be available in English and used in empirical research since the year 2000.

**Sources of evidence**: A search has been performed through Google Scholar and two other disciplinary bibliographies edited by international learned societies.

**Charting methods**: The items of each questionnaire are categorized based on their wordings, and thus independently from the conceptual models within which they have been developed.  Based on this categorization, various domains to which the items can be ascribed are identified (e.g. space, realism, agency, etc.) and psychological phenomena are linked to them (e.g. presence, social presence, narrative absorption, etc.).

**Results**: 308 items in 23 questionnaires have been found to have overlapping of wordings.

**Conclusions**: A list of the core aspects of presence, social presence, flow, and narrative absorption is presented, together with a critical selection of items suitable to measure each construct.

## Introduction

### Rationale

Experiences mediated by technology (e.g. printed books, screens, and virtual reality) are studied across a variety of disciplines, often with little cooperation. Different theorizations, models, and empirical tools have been developed, resulting in a fuzzy agglomerate of related and overlapping concepts, like presence ( Lombard *et al*., 2015), flow ( Csikszentmihalyi, 1990; Harmat *et al*., 2016), and narrative absorption ( Hakemulder *et al*., 2017). A scoping review is a suitable method to identify and summarize the core aspects of these various concepts, since they are currently obscured by the heterogeneity of disciplines investigating them. I surveyed the questionnaires most used in empirical research regarding this kind of psychological phenomena and I categorized the items in each questionnaire based on their wordings, thus independently from the conceptual models within which they have been developed. Overlapping concepts have been formulated in different fields according to specific disciplinary interests and based on knowledge within each field, this review focuses on how language is actually used in questionnaire items, rather than on how concepts are formulated top-down and associated with corresponding linguistic expressions that become items of a questionnaire.

### Objectives

The goal is to highlight similarities and overlaps between questionnaires’ items in order to identify which are the most relevant aspects of the psychological phenomena labelled as “presence,” “flow,” and “narrative absorption.” Based on this categorization, I suggested the domains to which each group of items can be ascribed (e.g. space, realism, agency, etc.) and I associated them to the respective psychological phenomena for which they are more frequently used (e.g. presence, social presence, narrative absorption, etc.).

## Methods

### Protocols and registration

I followed Arksey and O’Malley’s framework for scoping reviews ( Arksey & O’Malley, 2005), refined by Levac *et al*. (2010) and the Joanna Briggs Institute ( Peters *et al*., 2015). I reported findings following the PRISMA-ScR (Preferred Reporting Items for Systematic reviews and Meta-Analyses extension for Scoping Reviews) checklist ( Tricco *et al*., 2018).

### Eligibility criteria

The sources considered are questionnaires available in English, no year limit has been used. To be included in the review, questionnaires need to have been developed or used for research about one of the following media: VR, video game, film, book. I only included questionnaires measuring psychological states, not those measuring personality traits or broader psychological concepts (e.g. state empathy has been included, but not trait empathy). Validation and statistical reliability were not necessary criteria.

### Information sources

I performed the search in May 2020, using three sources: the aggregator Google Scholar, the bibliography of the International Society for the Empirical Study of Literature (IGEL), and the measurement guides provided by the International Society for Presence Research (ISPR). Additional useful comparisons of presence-related concepts can be found in Paiva de Oliveira *et al*., 2016; van Baren & IJsselsteijn (2004), and Skarbez *et al*. (2017); for narrative absorption and similar concepts, see Busselle & Bilandzic (2017); for games, see Reddy (2016).

### Search

The queries used in Google Scholar are: “presence questionnaire,” “immersion questionnaire,” “flow questionnaire,” “narrative questionnaire,” “narrative engagement,” “narrative absorption,” “narrative transportation.”

### Selection of sources of evidence

I obtained information about questionnaires directly from published articles and also from reviews included in Master theses or PhD dissertations. The criterion used to consider a questionnaire eligible as a source of evidence is its application in recent years: once I identified a questionnaire, I checked its use in research starting from the year 2000. I made this selection also with the help of a review of the questionnaires most used in VR research in the years 2016–17 ( Hein *et al.*, 2018).

### Data charting process

When multiple versions of a questionnaire were available, I considered only the most recent or shortest version, since this is likely to be an improvement over previous or longer versions, with respect to the goal of this scoping review. I then recorded each item of the data in a spreadsheet and manually annotated them.

### Data items

Being a data-driven bottom-up review, I did not define any specific variables *a priori*. Rather, I analyzed all questionnaires’ items. Among the total items in all the questionnaires studied, I only grouped and categorized the items for which I found close similarities and overlap of wordings.

### Critical appraisal of individual sources of evidence

From a preliminary screening, I found that some items inquire about more than one aspect of the target experience. During the analysis, I identified all such items and excluded them from the synthesis of results, in order to avoid confusion with respect to the aspect covered by each type of item.

### Synthesis of results

I compared the items of the selected questionnaires and grouped them according to similarities in the wordings used. For instance, the narrative absorption item “When I was finished with reading the story it felt like I had taken a trip to the world of the story” ( Kuijpers *et al*., 2014) strongly resembles the spatial presence item “After my experience of the displayed environment, I had a sense that I had returned from a journey” ( Lessiter *et al*., 2001). Once I have identified various clusters of items, I labelled each group and linked it to the most relevant psychological phenomenon. When items were already originally grouped in subdimension of the broader psychological construct, I used the subdimensions as guidance for the classification.

## Results

### Selection of sources of evidence

**Figure 1.**
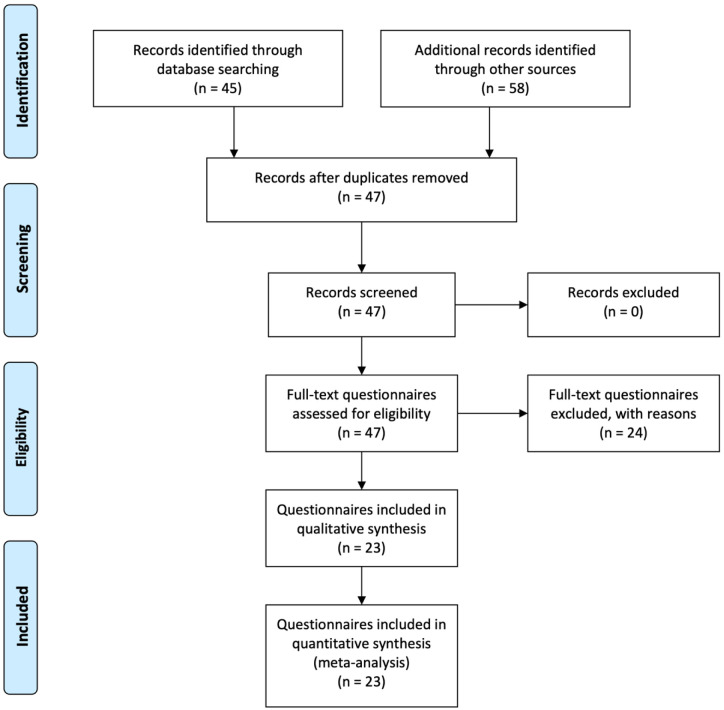
Flow diagram of the selection of sources process (adapted from Moher *et al.*, 2009).

The process of selection is outlined in Figure 1.

### Characteristics of sources of evidence

The questionnaires analyzed are listed in Table 1. Out of the 23 questionnaires included in the analysis, 8 have been developed to measure presence, 3 for flow, 6 for game immersion/engagement, and 6 for narrative phenomena (absorption, engagement, transportation, immersion, identification with characters, and empathy with characters).

**Table 1.** Questionnaires analyzed and categorized. Total number of items, n= 484.

	Questionnaire	Type	Total number of items	Number of selected items
1	Temple Presence Inventory (TPI) ( Lombard *et al*., 2000)	Presence	42	18
2	Slater, Usoh and Steed (SUS) ( Usoh *et al*., 2000)	Presence	6	3
3	Sense of Presence Inventory (ITC-SOPI) ( Lessiter *et al*., 2001)	Presence	44	23
4	Igroup Presence Questionnaire (IPQ) ( Schubert, 2003)	Presence	14	10
5	Networked Minds Social Presence Inventory (NMSPI) ( Harms & Biocca, 2004)	Presence	36	33
6	Presence Questionnaire, version 3 (PQ) ( Witmer *et al*., 2005)	Presence	29	10
7	Spatial Presence Experience Scale (SPES) ( Hartmann *et al*., 2016)	Presence	8	8
8	Multimodal Presence Scale (MPS) ( Makransky *et al*., 2017)	Presence	15	12
9	Flow Short Scale (FSS) ( Rheinberg, 2008)	Flow	13	8
10	EduFlow Scale (EFS) ( Heutte *et al*., 2014)	Flow	12	8
11	Reading Flow Short Scale (RFSS) ( Thissen *et al*., 2018)	Flow	8	6
12	EGameFlow (EGF) ( Fu *et al*., 2009)	Game/Flow	42	16
13	Immersion in the Narrative Game Questionnaire (INGQ) ( Qin *et al*., 2009)	Game	27	18
14	Game Engagement Questionnaire (GEQ) ( Brockmyer *et al*., 2009)	Game	19	12
15	User Engagement Scale (UES) ( O’Brien & Toms, 2013; Wiebe *et al*., 2014)	Game	28	15
16	Game Experience Questionnaire (GExQ) ( IJsselsteijn *et al*., 2013)	Game	40	26
17	Game Immersion Questionnaire (GIQ) ( Cheng *et al*., 2015)	Game	14	7
18	Transportation Scale ( Green & Brock, 2000)	Narrative	11	10
19	Identification Scale ( Cohen, 2001)	Narrative	10	9
20	Narrative Engagement Scale (NES) ( Busselle & Bilandzic, 2009)	Narrative	12	12
21	State Empathy Scale (SES) ( Shen, 2010)	Narrative	12	9
22	Story World Absorption Scale (SWAS) ( Kuijpers *et al*., 2014)	Narrative	18	17
23	Film Immersion Questionnaire (FIQ) ( Jennett *et al*., 2008; Rigby *et al*., 2019)	Narrative	24	18

### Critical appraisal within sources of evidence

Some items present a combination of more than one aspect, so I excluded them from the synthesis of the results in order to avoid confusion within each group of items. For instance, the item “I lose perceptions of time and the real world surrounding me, as if everything just stops” (Game Immersion Questionnaire, Cheng *et al.*, 2015) asks about the perception of both time and space. I also excluded items inquiring about some of the aspects identified when they have peculiar wordings that do not overlap with other items. Out of the total 484 items, 308 (64%) have close similarities and overlapping of wordings.

### Results of individual sources of evidence

Table 1 reports the number of items selected in each questionnaire.

### Synthesis of the results

The complete categorization of the questionnaire items can be found in the underlying data. A summary of the most frequent categories is reported in Table 2. Attention is undoubtedly the most relevant term for all the constructs considered, conceived as disregard for both thoughts and perceptions that are not part of the activity eliciting presence, flow, or absorption. Similarly, a distorted perception of time is in many cases considered to be a sign of the occurrence of all the considered phenomena. With respect to categories specific to each concept, spatial presence is characterized by items related to space, agency, and a comparison with reality not mediated by technology. Social presence is characterized by the same categories that are relevant for spatial presence (space and agency) but in relation to the existence of other agents; additionally, some kind of cognitive attention to the other and emotional arousal elicited by them are also frequent. Flow is specifically characterized by the perception of a sense of challenge. Narrative absorption is characterized by a comparison with non-mediated reality (in terms of vividness of imagery), by an easy comprehension of content, and by emotions and thoughts anticipating possible outcomes (suspense). Lastly, there are two groups of items explicitly asking about the user’s perception of involvement/engagement or absorption/immersion.

**Table 2.** Categorization of items (n = 308) from presence, flow, game, and narrative questionnaires.

Total items	Scales with item	Item type	Category	Main psychological phenomenon
23	11	Attention (no external thoughts)	Attention	Attention
17	9	Attention (no external perceptions)		
18	11	Time distortion	Time	–
17	9	“Being there” (feelings and perceptions, not thoughts)	Space	Spatial presence
8	5	Realities overlapping		
6	3	Closeness of story world		
7	6	Return to reality		
5	5	Being part of the action (also partly overlaps with "being there")		
10	5	Possibility of action in space	Agency	
6	4	Control of content		
5	3	Control of medium		
9	6	Naturalness/fluency of medium use		
14	6	Perceived realism	Comparison	
5	2	Attention to another agent	Attention	Social presence
5	4	Co-location with another agent	Space	
24	4	Mind reading	Cognition	
5	2	Behavioural response to another agent	Agency	
13	7	Matching of another agent 's emotions	Emotion	
4	3	Feelings for another agent		
6	5	Connection with another agent	Emotion/ Cognition	
16	6	Understanding of another agent (perspective taking, cognitive empathy)	Cognition	
12	7	Challenge	Cognition	Flow
8	4	Vividness of imagery	Comparison	Narrative absorption
14	7	Comprehension of content	Comprehension	
9	6	Suspense/anticipation	Emotion/ Cognition	
18	10	Emotional response to medium/content	Emotion	Emotional impact
14	7	Explicit use of involvement/engagement terms	Metaphor	–
10	9	Explicit use of absorption/immersion terms		

## Discussion

### Summary of evidence

In all questionnaires, the most frequently recurring items concern attention and the sense of time. The isolation from external thoughts and perceptions is the main characteristic of presence-related phenomena, and such disconnection from stimuli unrelated to the undergoing experience probably leads to an alteration of the sense of time. Despite the evolution towards broad psychological conceptions of presence ( Baños *et al*., 2000; Lee, 2004; Riva *et al*., 2015), a review ( Hein *et al*., 2018) of the psychometric questionnaires used in VR research in the years 2016–17 found that the most used one is the Presence Questionnaire ( Witmer & Singer, 1998), which heavily focuses on visual realism and naturalness of interaction. However, the broadest and most protracted collective effort aimed at clarifying how to measure presence ( Hartmann *et al*., 2016; Vorderer *et al*., 2004) has excluded realism from the subdimensions of presence, keeping only “self-location” and “possible action” as core dimensions. Indeed, these two categories seem to be the two really specific to presence, since a comparison with non-mediated reality is also relevant for the “imagery” category, which concerns items related to narrative absorption. Inquiring about the vividness of imagery or about the realism of a VR scene is a way to check how similar the imagined/mediated experience is to a non-mediated one. Both realism and vivid imagery are outcomes that can be associated with presence, but they are not particularly helpful to explain the underlying psychological processes that bring to the emergence of a sense of presence.

Many questionnaires also take into account the possibility that perceiving the existence of other agents can affect our sense of presence or, more broadly, that we can have intense experiences when interacting with others or following their actions. With a growing degree of complexity, such perception goes from merely noticing the existence of others, to interacting with them, to emotional and cognitive ways of responding to and understanding others’ mental states. These groups of items, which I have associated with the concept of *social presence*, occur often together with spatial presence items and seem to entail it as the basis on top of which they can emerge. Indeed, they are all different expressions of a self-other relationship and can be conceptualized as forms of presence in co-participation. Analogously, questionnaires about flow experiences include items that I have here associated with spatial presence – and in some cases also items related to social presence – plus a specific group of questions regarding the perception of an experience as challenging. Similar wordings can be also found in items of narrative and game questionnaires.

Items that I specifically associated with the concept of narrative absorption regard imagery, the feeling of suspense triggered by the narrated events, and the comprehension of the content of the story, an aspect which can be connected to the sense of challenge of flow experiences, since the right match between the complexity of a story and the cognitive skills of the audience is relevant for narrative absorption. It is worth noting that questionnaires investigating narrative absorption include these three groups of items but also items related to spatial presence and social presence (with characters of a story), which can be considered subdimensions of narrative absorption. Given their metaphorical nature, items explicitly asking whether an experience elicited involvement, engagement, immersion, or absorption are not particularly useful for describing the psychological processes activated during the experiences they aim at qualifying. Moreover, the adjective “immersive” is used in VR research as a technical attribute of the medium – consistently with Sheridan seminal definition ( Sheridan, 1992) – whereas in game and narrative studies it is a quality of the player or reader’s experience ( Jennett *et al*., 2008; Ryan, 2015; Stockwell, 2019).

Another popular but quite heterogeneous group of questions concerns the emotional impact of mediated experiences. Ten questionnaires investigate this aspect in slightly different ways, so it is hard to say whether emotional impact is a component of any of the presence-related phenomena or a secondary effect elicited by them.

The recognition presented can be used to reflect on the extent to which wording similarities among items from different questionnaires actually result from similarities between the underlying conceptualizations. One possible outcome is a cross-disciplinary systematization of concepts, suggesting viable options for an interdisciplinary agreement about the core aspects of the psychological states elicited by mediated experiences. To sum up, attention and time distortion are common to all the considered phenomena, and spatial presence (space and agency) is the phenomenon with the narrowest scope, the core. Social presence and narrative absorption are phenomena of increasingly broader scope, each of them including the listed phenomena of narrower scope. Flow is a concept transversal to the other three, being more related to the balance between a person’s skills and the complexity of the stimulus, rather than to a specific psychological dimension.

Following the above-mentioned strategy, in Table 3 I summarized the conceptual overlaps that can be inferred from the similarities between items, and I recommend the subdimension that best correspond to the various groups of items. Additionally, in Table 4, I present a selection of items that best correspond to the categories identified by my inductive process. The use of such items to measure presence, social presence, and narrative absorption can help to achieve a more solid epistemic comparability among research on these phenomena. In order to benefit from previous statistical validations, in case of similarities, I gave preference to items coming from the same questionnaire. Depending on the task/content with which the participants are engaging, only a part of these items may be relevant.

**Table 3.** Selection of questionnaire subdimensions recommended to achieve a more solid epistemic comparability among research on presence, social presence, and narrative absorption.

Item type	Category	Recommended questionnaire subdimension	Main psychological phenomenon
Attention (no external thoughts)	Attention	NES by Busselle & Bilandzic (2009) – “Attentional focus”	Attention
Attention (no external perceptions)		PQ v.3 by Witmer *et al*. (2005) – “Adaptation/Immersion” / FIQ by Rigby *et al*. (2019) – “ Real-world Dissociation”	
Time distortion	Time	Various	–
“Being there” (feelings and perceptions, not thoughts)	Space	SPES by Hartmann *et al*. (2016) – “Self- location”	Spatial presence
Realities overlapping			
Closeness of story world			
Return to reality			
Being part of the action (also partly overlaps with "being there")			
Possibility of action in space	Agency	SPES by Hartmann *et al*. (2016) – “Possible action”	
Control of content			
Control of medium			
Naturalness/fluency of medium use			
Attention to another agent	Attention	NMSPI by Harms & Biocca (2004) – “Perceived Attentional Engagement”	Social presence
Co-location with another agent	Space	MPS by Makransky *et al*. (2017) – “Social presence”	
Mind reading	Cognition		
Behavioural response to another agent	Agency	NMSPI by Harms & Biocca (2004) – “Perceived Behavioural Interdependence”	
Matching of another agent 's emotions	Emotion	NMSPI by Harms & Biocca (2004) – “Perceived Emotional Contagion” / SES by Shen (2010) – “Affective empathy”	
Feelings for another agent			
Connection with another agent	Emotion/Cognition		
Understanding of another agent (perspective taking, cognitive empathy)	Cognition	NMSPI by Harms & Biocca (2004) – “Perceived Comprehension” / SES by Shen (2010) – “Cognitive empathy”	
Challenge	Cognition	RFSS by Thissen *et al*. (2018) – “Absorption”	Flow
Vividness of imagery	Comparison	SWAS by Kuijpers *et al*. (2014) – “Mental imagery”	Narrative absorption
Comprehension of content	Comprehension	NES by Busselle & Bilandzic (2009) – “Narrative understanding”	
Suspense/anticipation	Emotion/Cognition	Transportation Scale by Green & Brock (2000) – “Transportation”	

**Table 4.** Selection of questionnaire items (with minimal adaptation) recommended to achieve a more solid epistemic comparability among research on presence, social presence, and narrative absorption. (R = reverse scored).

	Item	Item type	Recommended questionnaire subdimension	Main psychological phenomenon
1	While [task/content] I found myself thinking about other things. [R]	Attention (no external thoughts)	NES by Busselle & Bilandzic (2009) – “Attentional focus”	Attention
2	I had a hard time keeping my mind on the [task/content]. [R]			
3	I was able to concentrate very well on [task/content] rather than on the mechanisms used to [perform/represent] that [task/content].	Attention (no external perceptions)	PQ v.3 by Witmer *et al*. (2005) – “Adaptation/Immersion”	
4	I didn’t notice events taking place around me.		FIQ by Rigby *et al*. (2019) – “Real- world Dissociation”	
5	I lost track of time.	Time distortion	Various	–
6	I felt like I was actually there in the environment of the presentation.	Self-location	SPES by Hartmann *et al*. (2016) – “Self-location”	Spatial presence
7	It seemed as though I actually took part in the action of the presentation.			
8	It was as though my true location had shifted into the environment in the presentation.			
9	I felt as though I was physically present in the environment of the presentation.			
10	The objects in the presentation gave me the feeling that I could do things with them.	Possible action	SPES by Hartmann *et al*. (2016) – “Possible action”	
11	I had the impression that I could be active in the environment of the presentation.			
12	I felt like I could move around among the objects in the presentation.			
13	It seemed to me that I could do whatever I wanted in the environment of the presentation.			
14	I paid close attention to [other agent/s].	Attention to another agent	NMSPI by Harms & Biocca (2004) – “Perceived Attentional Engagement”	Social presence
15	I was easily distracted from [other agent/s] when other things were going on. [R]			
16	I felt like I was in the presence of someone else while [task/ content].	Co-location with another agent	MPS by Makransky *et al*. (2017) – “Social presence”	
17	I felt that the [other agent/s] in [place] were aware of my presence.	Mind reading		
18	The [other agent/s] in [place] appeared to be sentient (conscious and alive) to me.			
19	My actions were often dependent on [other agent/s’] actions.	Perceived Behavioural Interdependence	NMSPI by Harms & Biocca (2004) – “Perceived Behavioural Interdependence”	
20	My behavior was often in direct response to [other agent/s’] behavior.			
21	What [other agent/s] did often affected what I did.			
22	I was sometimes influenced by [other agent/s’] moods.	Affective empathy	NMSPI by Harms & Biocca (2004) – “Perceived Emotional Contagion”	
23	I experienced the same emotions as the [other agent/s] while [task/content].		SES by Shen (2010) – “Affective empathy”	
24	I could feel the [other agent/s’] emotions.			
25	I was able to understand what [other agent/s’] meant.	Understanding of another agent (perspective taking, cognitive empathy)	NMSPI by Harms & Biocca (2004) – “Perceived Comprehension”	
26	I can see the [other agent/s’] point of view.		SES by Shen (2010) – “Cognitive empathy”	
27	I can understand what the [other agent/s’] was going through.			
28	I felt optimally challenged while [task/content].	Challenge	RFSS by Thissen *et al*. (2018) – “Absorption”	Flow
29	When I was reading the story, I had an image of the main character in mind.	Vividness of imagery	SWAS by Kuijpers *et al*. (2014) – “Mental imagery”	Narrative absorption
30	When I was reading the story, I could see the situations happening in the story being played out before my eyes.			
31	I could imagine what the world in which the story took place looked like.			
32	At points, I had a hard time making sense of what was going on in the story. [R]	Comprehension of content	NES by Busselle & Bilandzic (2009) – “Narrative understanding”	
33	I wanted to learn how the story ended.	Suspense/anticipation	Transportation Scale by Green & Brock (2000) – “Transportation”	

### Limitations

Categorizing only 308 items, out of the total 484 found in the sampled questionnaires, this scoping review may have missed some aspects of presence and related concepts that are important to grasp the nuances of the phenomenal experience that may be specific to certain media. However, by focusing on items showing a recurring intersubjective agreement between researchers and disciplines, I think I have successfully identified and summarized the core aspects of the surveyed phenomena. However, it is worth remembering that the employment of measurement tools should always be justified by theoretical reflection and empirical validation. A scoping review is an aid for the systematization of knowledge, but it also produces new knowledge that requires further scrutiny and methodological testing before it can be deployed into experimental settings.

## Conclusions

The categorization proposed here can be used to further refine existing questionnaires and possibly encourage a convergence of different disciplines towards a use of the same items, so that insight coming from different fields could be used for the advancement of knowledge in specific areas. For instance, empirical research on narrative could benefit from using existing items for presence and social presence, without “reinventing the wheel” and focusing rather on refining how to measure dimensions like suspense and imagery. Moreover, a shared agreement on basic items will enable better and more informative meta-analyses, as well as comparative media studies, a kind of research that is strongly relevant for all the disciplines that I mentioned here, since only a comparison between experiences with different media can help to account for the specificity of presence and related phenomena.

## Data availability

### Underlying data

OSF: Presence, flow, and narrative absorption questionnaires: a scoping review

https://doi.org/10.17605/OSF.IO/RBZ8G ( Pianzola, 2021)

This project contains the following underlying data:

- scoping_review_data_2021-02-26.xlsx (Human-readable version containing the 23 selected questionnaires with color coding of the items and summary model)
- scoping_review_data_2021-02-26.csv (Machine-readable version containing the 23 selected questionnaires with the respective annotations for each item)

Data are available under the terms of the Creative Commons Zero "No rights reserved" data waiver (CC0 1.0 Public domain dedication).

### Extended data

***Reporting guidelines***

OSF: PRISMA-ScR checklist for ‘Presence, flow, and narrative absorption questionnaires: a scoping review’.

https://doi.org/10.17605/OSF.IO/RBZ8G ( Pianzola, 2021)

Data are available under the terms of the Creative Commons Zero “No rights reserved” data waiver (CC0 1.0 Public domain dedication).

## References

[ref-1] Arksey H O’Malley L : Scoping studies: Towards a methodological framework. *Int J Soc Res Methodol.* 2005;8(1):19–32. 10.1080/1364557032000119616

[ref-2] Baños RM Botella C Garcia-Palacios A : Presence and Reality Judgment in Virtual Environments: A Unitary Construct? *Cyberpsychol Behav.* 2000;3(3):327–335. 10.1089/10949310050078760

[ref-3] Brockmyer JH Fox CM Curtiss KA : The development of the Game Engagement Questionnaire: A measure of engagement in video game-playing. *J Exp Soc Psychol.* 2009;45(4):624–634. 10.1016/j.jesp.2009.02.016

[ref-4] Busselle R Bilandzic H : Measuring narrative engagement. *Media Psychology.* 2009;12(4):321–347. 10.1080/15213260903287259

[ref-5] Busselle R Bilandzic H : Beyond metaphors and traditions. Exploring the conceptual boundaries of narrative engagement.In F. Hakemulder, M. M. Kuijpers, E. S. Tan, K. Bálint, & M. M. Doicaru (Eds.): *Narrative Absorption.*John Benjamins,2017;11–27. 10.1075/lal.27.02bil

[ref-6] Cheng MT She HC Annetta LA : Game immersion experience: Its hierarchical structure and impact on game-based science learning. Impact of immersion on learning. *J Comput Assist Learn.* 2015;31(3):232–253. 10.1111/jcal.12066

[ref-7] Cohen J : Defining Identification: A Theoretical Look at the Identification of Audiences with Media Characters. *Mass Commun Soc.* 2001;4(3):245–264. 10.1207/S15327825MCS0403_01

[ref-8] Csikszentmihalyi M : Flow: The psychology of optimal experience.Harper Collins,1990. Reference Source

[ref-10] Fu FL Su RC Yu SC : EGameFlow: A scale to measure learners’ enjoyment of e-learning games. *Comput Educ.* 2009;52(1):101–112. 10.1016/j.compedu.2008.07.004

[ref-11] Green MC Brock TC : The role of transportation in the persuasiveness of public narratives. *J Pers Soc Psychol.* 2000;79(5):701–721. 10.1037//0022-3514.79.5.701 11079236

[ref-12] Hakemulder F Kuijpers MM Tan ES : Narrative Absorption.John Benjamins,2017. 10.1075/lal.27

[ref-13] Harmat L Andersen FØ Ullén FØ : Flow Experience: Empirical Research and Applications.Springer International Publishing,2016. 10.1007/978-3-319-28634-1

[ref-14] Harms C Biocca F : Internal Consistency and Reliability of the Networked Minds Measure of Social Presence.In M. Alcañiz & B. Rey (Eds.): *Seventh Annual International Workshop: Presence 2004*.2004. Reference Source

[ref-15] Hartmann T Wirth W Schramm H : The Spatial Presence Experience Scale (SPES): A Short Self-Report Measure for Diverse Media Settings. *J Media Psychol.* 2016;28(1):1–15. 10.1027/1864-1105/a000137

[ref-16] Hein D Mai C Hußmann H : The usage of presence measurements in research: A review. *Proceedings of the 17th Conference of the International Society for Presence Research (ISPR).* 2018. Reference Source

[ref-17] Heutte J Fenouillet F Boniwell I : Optimal learning experience in digital environments: Theoretical concepts, measure and modelisation. *Digital Learning in 21st Century Universities*. Georgia Institute of Technology, Atlanta,2014. Reference Source

[ref-18] IJsselsteijn W de Kort YAW Poels K : The Game Experience Questionnaire. Technische Universiteit Eindhoven.2013. Reference Source

[ref-19] Jennett C Cox AL Cairns P : Measuring and defining the experience of immersion in games. *Int J Hum Comput Stud.* 2008;66(9):641–661. 10.1016/j.ijhcs.2008.04.004

[ref-101] Kuijpers MM Hakemulder F Tan ES : Exploring absorbing reading experiences: Developing and validating a self-report scale to measure story world absorption. *Scientific Study of Literature.* 2014;4(1):89–122. 10.1075/ssol.4.1.05kui

[ref-21] Lee KM : Presence, Explicated. *Commun Theory.* 2004;14(1):27–50. 10.1111/j.1468-2885.2004.tb00302.x

[ref-102] Lessiter J Freeman J Keogh E : A Cross-Media Presence Questionnaire: The ITC-Sense of Presence Inventory. *Presence (Camb).* 2001;10(3):282–297. 10.1162/105474601300343612

[ref-20] Levac D Colquhoun H O’Brien KK : Scoping studies: Advancing the methodology. *Implement Sci.* 2010;5(1):69. 10.1186/1748-5908-5-69 20854677 PMC2954944

[ref-23] Lombard M Biocca F Freeman J : Immersed in Media: Telepresence Theory, Measurement & Technology.Springer,2015. 10.1007/978-3-319-10190-3

[ref-22] Lombard M Ditton TB Crane D : Measuring Presence: A Literature-Based Approach to the Development of a Standardized Paper-and-Pencil Instrument. *Presence 2000: The Third International Workshop on Presence.* 2000. Reference Source

[ref-24] Makransky G Lilleholt L Aaby A : Development and validation of the Multimodal Presence Scale for virtual reality environments: A confirmatory factor analysis and item response theory approach. *Comput Human Behav.* 2017;72:276–285. 10.1016/j.chb.2017.02.066

[ref-25] Moher D Liberati A Tetzlaff J : Preferred Reporting Items for Systematic Reviews and Meta-Analyses: The PRISMA Statement. *PLoS Med.* 2009;6(7):e1000097. 10.1371/journal.pmed.1000097 19621072 PMC2707599

[ref-26] O’Brien HL Toms EG : Examining the generalizability of the User Engagement Scale (UES) in exploratory search. *Inf Process Manag.* 2013;49(5):1092–1107. 10.1016/j.ipm.2012.08.005

[ref-9] Paiva de Oliveira R Calsavara Paiva de Oliveira D Fernandes Tavares T : Measurement Methods for Phenomena Associated with Immersion, Engagement, Flow, and Presence in Digital Games. *SBC - Proceedings of SBGames 2016.* 2016;127–135. Reference Source

[ref-27] Peters MDJ Godfrey CM Khalil H : Guidance for conducting systematic scoping reviews. *Int J Evid Based Healthc.* 2015;13(3):141–146. 10.1097/XEB.0000000000000050 26134548

[ref-150] Pianzola F : Presence, flow, and narrative absorption questionnaires: a scoping review (supplementary material).2021. 10.17605/OSF.IO/RBZ8G PMC10446018 37645174

[ref-28] Qin H Patrick Rau PL Salvendy G : Measuring Player Immersion in the Computer Game Narrative. *Int J Hum-Comput Int.* 2009;25(2):107–133. 10.1080/10447310802546732

[ref-29] Reddy GSH : Empirical Investigation on Measurement of Game Immersion using Real World Dissociation Factor. Blekinge Institute of Technology,2016. Reference Source

[ref-30] Rheinberg F : Intrinsic motivation and flow-experience.In H. Heckhausen & J. Heckhausen (Eds.), *Motivation and Action.*Cambridge University Press,2008;323–348. 10.1017/CBO9780511499821.014

[ref-31] Rigby JM Brumby DP Gould SJJ : Development of a Questionnaire to Measure Immersion in Video Media: The Film IEQ. *Proceedings of the 2019 ACM International Conference on Interactive Experiences for TV and Online Video - TVX ’ 19.* 2019;35–46. 10.1145/3317697.3323361

[ref-32] Riva G Mantovani F Waterworth EL : Intention, Action, Self and Other: An Evolutionary Model of Presence. In M. Lombard, F. Biocca, J. Freeman, W. IJsselsteijn, & R. J. Schaevitz (Eds.). *Immersed in Media.* 2015;73–99. 10.1007/978-3-319-10190-3_5

[ref-33] Ryan ML : Narrative as virtual reality 2: Revisiting immersion and interactivity in literature and electronic media.(Second edition). Johns Hopkins University Press,2015. Reference Source

[ref-34] Schubert TW : The sense of presence in virtual environments: A three-component scale measuring spatial presence, involvement, and realness. *Zeitschrift Für Medienpsychologie.* 2003;15(2):69–71. 10.1026//1617-6383.15.2.69

[ref-35] Shen L : On a scale of state empathy during message processing. *West J Commun.* 2010;74(5):504–524. 10.1080/10570314.2010.512278

[ref-36] Sheridan TB : Musings on Telepresence and Virtual Presence. *Presence (Camb).* 1992;1(1):120–126. 10.1162/pres.1992.1.1.120

[ref-37] Skarbez R Brooks FP Jr Whitton MC : A Survey of Presence and Related Concepts. *ACM Comput Surv.* 2017;50(6):1–39. 10.1145/3134301

[ref-38] Stockwell P : Immersion and Emergence in Children’s Literature. In Neurohr, B. and Stewart-Shaw, L. (Eds.). *Experiencing Fictional Worlds.* John Benjamins,2019;15–32. 10.1075/lal.32.02sto

[ref-39] Thissen BAK Menninghaus W Schlotz W : Measuring Optimal Reading Experiences: The Reading Flow Short Scale. *Front Psychol.* 2018;9:2542. 10.3389/fpsyg.2018.02542 30618963 PMC6300572

[ref-40] Tricco AC Lillie E Zarin W : PRISMA Extension for Scoping Reviews (PRISMA-ScR): Checklist and Explanation. *Ann Intern Med.* 2018;169(7):467–473. 10.7326/M18-0850 30178033

[ref-41] Usoh M Catena E Arman S : Using Presence Questionnaires in Reality. *Presence (Camb).* 2000;9(5):497–503. 10.1162/105474600566989

[ref-42] van Baren J IJsselsteijn W : Measuring Presence: A Guide to Current Measurement Approaches [OmniPres project IST-2001-39237].2004. Reference Source

[ref-43] Vorderer P Wirth W Gouveia FR : MEC Spatial Presence Questionnaire (MEC- SPQ): Short Documentation and Instructions for Application. Project Presence: MEC (IST-2001-37661). 2004.

[ref-44] Wiebe EN Lamb A Hardy M : Measuring engagement in video game-based environments: Investigation of the User Engagement Scale. *Comput Human Behav.* 2014;32:123–132. 10.1016/j.chb.2013.12.001

[ref-46] Witmer BG Jerome CJ Singer MJ : The Factor Structure of the Presence Questionnaire. *Presence (Camb).* 2005;14(3):298–312. 10.1162/105474605323384654

[ref-45] Witmer BG Singer MJ : Measuring Presence in Virtual Environments: A Presence Questionnaire. *Presence (Camb).* 1998;7(3):225–240. 10.1162/105474698565686

