# Supplementary material for: Presence, flow, and narrative absorption questionnaires: a scoping review
Source: Open Res Eur. 2021 Nov 4;1:11. Originally published 2021 Mar 24. [Version 2] doi: 10.12688/openreseurope.13277.2 (PMC10446018; doi:10.12688/openreseurope.13277.2)
Supplement: Peer review history

# Peer review: Reviewer response for version 2

**DOI:** 10.21956/openreseurope.15388.r29719

**Published:** 2022-08-04

**Authors:** Shawn Douglas

**Affiliations:** 1 University of Alberta, Edmonton, Canada

This article represents an attempt to survey and synthesize the conceptually related but practically disparate fields of immersion, flow, presence and narrative absorption. The focus is on an analysis of the use of language in questionnaire development for these constructs, in order to better understand their shared higher order structure.

 This is such a fantastic article for its ability to synthesize the base information needed to determine the kind of questionnaire that may be required, or could be useful, for a lot of basic research in the articulated fields. I wish that this was available when I first started my research in narrative absorption! I hope I can add something meaningful beyond the other two excellent reviews.

 I just have a few notes for minor revisions or future considerations:

1. There’s a slight contradiction in the eligibility criteria section, “available in English, no year limit has been used.” You do mention a year limit of 2000 in the abstract.
2. I’m also interested in the data charting process. For the use of the shortest version of the questionnaire, is there any documentation for what may be lost? There is almost certainly something lost in many questionnaires for deciding to use the short version over the long version, is there anything of note here?
3. For “data items”, was “close similarities” determined through inter-rater standards, or just what looked “close”? Perhaps this is just a nuance of the scoping method and can be ignored.
4. For “critical appraisal of individual sources of evidence”, is there any affordance for bivalent or complex experiences? Might this be included in a future study?
5. Were all removed articles (or questionnaires - n=24) removed because of complexity? It would be useful to have a better sense of why these articles were removed.
6. This is a fantastic first step, but I would love to see this article have at least one follow-up article – a tracing of theory in relation to linguistic use, as Christoph Klimmt points out, but it would also be useful to see how these concepts align with other pragmatic considerations (style of study, goal of study, etc.). This would not only make it easier for researchers to know which questionnaire might work best for their needs, but it would also allow for an easier way to branch out into related, but perhaps slightly different methodologies. The only reason I say, "partly" for "Are the conclusions drawn adequately supported by the results presented in the review?" is that I would like to see a little more discussion regarding future studies for how this work may be built upon and made into a study that makes that extra connection with practical use and theory.

Are sufficient details of the methods and analysis provided to allow replication by others?

Yes

Are the rationale for, and objectives of, the Systematic Review clearly stated?

Yes

Is the statistical analysis and its interpretation appropriate?

Yes

Are the conclusions drawn adequately supported by the results presented in the review?

Partly

Reviewer Expertise:

Narrative absorption; metaphor; psychometrics.

I confirm that I have read this submission and believe that I have an appropriate level of expertise to confirm that it is of an acceptable scientific standard.

# Peer review: Reviewer response for version 1

**DOI:** 10.21956/openreseurope.14347.r27020

**Published:** 2021-06-01

**Authors:** Christoph Klimmt

**Affiliations:** 1 Department of Journalism and Communication Research, Hanover University of Music, Drama and Media, Hanover, Germany

The motivation behind Federico Pianzola’s analysis is the enormous number of questionnaires and scales that have been developed across various fields in which scholars investigate media users' state of being 'captured‘, 'absorbed‘, 'transported‘ or 'involved‘ by a message and / or its delivery technology. Because much media innovation aims at improving or intensifying such user experiences, thematic research is thriving and very productive. As a consequence, a hard-to-overview landscape of empirical measures has evolved, and oftentimes, these measures obviously utilize similar-sounding questions and items for assessing (presumably) different concepts. Pianzola offers a scoping review of the items used in some 20+ such measures to investigate the extent to which such "overlaps" occur, to gain a better orientation of the measures available, and to derive recommendations about which measure to use for which concept.

 The author’s goal of serving an interdisciplinary research community that is confused by the many different published questionnaires with orientation knowledge is relevant and laudable, and the overview of measures, items and linguistic similarities among items is highly valuable. However, from a psychological-methodological point of view, the idea of scoping the items of different questionnaires *across* and *independent of their* theoretical concepts, is a kind of "reverse engineering“ – because Pianzola tacitly tries to build an integrative *theory* of media absorption by synthesizing the *measures* used for different concepts. This does, in my humble view, not work at all.

 First, the key reason why there are so many different questionnaires and measures out there is that there are so many concepts and different elaborations of the same concepts (e.g., there is a lot of competing understandings of "Presence"), and various author teams have tried to establish their conceptual understanding and an according measure. The large number of measures available on the market is thus a consequence of theoretical diversity (and also some chaos) – a normal situation in the social sciences in which there is no standard definition of entities, variables, or phenomena as it can be found in natural (hard) sciences. For instance, in social psychology, a huge number of "theories" is circulating, many of which differ from each other in similarly small nuances as conceptualizations of "Presence" differ in the current context. The multiplicity of similar concepts and of elaborations of the same concept is a notorious problem, and Pianzola is right that in the case of media absorption, in which many scholars from diverse fields are interested, the resulting complexity and chaos is particularly undesirable. – But this chaos cannot be resolved by distilling some 'optimal' choice of questionnaire items or scales from the abundance of available measures.

 This is because measures in psychology are developed *based on their theoretical foundation*. Using a specific conceptualization of, say, Spatial Presence, one author team has created their items to measure Presence and has done (more or less, high- or less-high-quality) research to validate their measure (against the background of their theory). It is certainly NOT the case that all research teams who have developed a questionnaire of Spatial Presence had the SAME basic conceptual understanding of Spatial Presence in mind when they crafted their items. It is therefore misleading that Pianzola writes "this review focuses on how language is actually used in questionnaire items, rather than on how concepts are formulated top-down and arbitrarily associated with corresponding linguistic expressions that become items of a questionnaire" – no author team would accept the assumption that they have "arbitrarily" linked item wordings to a concept; on the contrary, most authors have invested much thinking in finding item wordings that match their conceptual understanding. As a consequence, Pianzola’s (implicit) idea that we can reshuffle items from different measures to come up with best-choice approaches to assess certain concepts or conceptual dimensions disconnects each questionnaire from its individual theoretical base. This is not how social-scientific measurement works – it must always be "theory first – measurement second". Pianzola, however, disregards theoretical differences and suggests a kind of meta-measures based on wording similarities. Such wording similarities among items from different questionaires, however, actually result from similarities between the underlying conceptualizations, and maybe in some cases, from poor operationalizations (i.e., items badly formulated so that they accidentally overlap with items validly designed to measure something profoundly different).

 So I express a warning not to step over the necessity of theoretical integration and synthesis by simply searching for "good measures" based on linguistic similarity analysis. Measures only "work" within the context of their underyling theoretical substance, and this substance differs between all questionnaires, which cannot be ignored when comparing (or combining) measures. Hence, for epistemological reasons, the main objective that Pianzola is pursuing, cannot (and should not) be achieved.

 But the author’s review of the many similar-yet-different measures of the many similar-yet-(maybe?)-different concepts is nevertheless of great utility! First, the mere listing of available measures and the accompanying descriptions help to maintain an orientation of the existing diversity. And second, this scoping review must be understood by many scholars active in the field as a reminder that the notorious diversity and chaos that results from competing conceptualizations and individual desires to establish one’s own theory and measure (in spite of the existence of many other similar theories and measures) is a huge problem. The precise analysis of which items and questionnaires display which kind of linguistic overlap in spite of (alleged) conceptual discrepancies is a great contribution by Pianzola, because it helps to detect those spots in media absorption research that seem to require particular effort of theoretical re-thinking, integration, and synthesis. Practically speaking, scholars may refer to this scoping review when articulating (and justifying) their individual decision to use certain conceptualizations (and according measures) in their specific study, thus acknowledging the undesirable plurality of available concepts and measures and making an informed selection decision at the same time. So I encourage the author of this paper and all readers to reflect on the theory-measurement-relationship and to develop conclusions on how the highly diverse, cross-disciplinary research on media absorption phenomena can cope with the theoretical-methodological diversity and still maintain strong standards of excellence in social-scientific (self-report) measurement.

 Clearly, this question is a key challenge that hopefully will inspire many young scholars to work towards greater theoretical integration, harmony, and parsimony. Based on my own experience with measuring user states that occur during media exposure but that are only assessed cumulatively after exposure, I end with the recommendation of not being too ambitious regarding the ability of assessing (theoretically) fine-grained differences in experience this way. Language that is comprehensible to laypersons is hardly capable to make experiential nuances (e.g., between "transportation" and "spatial presence") distinguishable. So one issue that Pianzola inspired me to reflect on is this specific aspect of the theory-measurement-relationship: Small theoretical discrepancies may simply not be possible to translate validly and precisely into item wording differences. So maybe media absorption research is in need of a new pragmatism in measuring concepts *in spite of* conceptual diversities. Authors of (slightly) differing concepts should thus feel invited to team up and debate whether they can agree on an integrated measure that would fit *both* their concepts. Now that would be a pathway of "bottom-up" integration of measures and hence a potentially viable way to move forward into the direction that Pianzola has shown to us.

Are sufficient details of the methods and analysis provided to allow replication by others?

Yes

Are the rationale for, and objectives of, the Systematic Review clearly stated?

Yes

Is the statistical analysis and its interpretation appropriate?

Yes

Are the conclusions drawn adequately supported by the results presented in the review?

Partly

Reviewer Expertise:

NA

I confirm that I have read this submission and believe that I have an appropriate level of expertise to confirm that it is of an acceptable scientific standard, however I have significant reservations, as outlined above.

# Response

**Published:** 2021-10-29

**Authors:** Federico Pianzola

**Affiliations:** University of Milan Bicocca, Italy

I am very grateful to Christoph Klimmt for his thorough commentary. I am very glad to see that we agree on the main epistemological goal of my scoping review:

- *The precise analysis of which items and questionnaires display which kind of linguistic overlap in spite of (alleged) conceptual discrepancies is a great contribution by Pianzola, because it helps to detect those spots in media absorption research that seem to require particular effort of theoretical re-thinking, integration, and synthesis. Practically speaking, scholars may refer to this scoping review when articulating (and justifying) their individual decision to use certain conceptualizations (and according measures) in their specific study, thus acknowledging the undesirable plurality of available concepts and measures and making an informed selection decision at the same time.*

Klimmt also rightfully points out that the main objective of my work may be mistaken for a ready-to-use optimal choice of questionnaire items to be employed to measure presence, social presence, and narrative absorption. In light of this, I have now reworded the presentation of my suggestions, stressing that they can be a useful schema to foster more awareness about advancements in other disciplinary fields. Hopefully, this will also lead scholars to aim for epistemic comparability, in the spirit of open science. Indeed, participating in a collective scientific effort to improve the understanding of what presence and narrative absorption are is more valuable than pursuing a finely nuanced grasping of human experience via the use of rigidly structured proxies like psychometric questionnaires. I agree that social-scientific measurement must always be “theory first – measurement second” and I hope this scoping review will help to refine theories before a selection of questionnaire items is made. I have now made this explicit in the Discussion and Conclusion.

# Peer review: Reviewer response for version 1

**DOI:** 10.21956/openreseurope.14347.r26866

**Published:** 2021-05-24

**Authors:** Tiago Fernandes Tavares

**Affiliations:** 1 School of Electrical and Computer Engineering, University of Campinas, Campinas, Brazil

The article provides a systematic overview of presence, flow, and narrative absorption questionnaires available in the literature.

 I believe this is an important work towards understanding how these concepts - which are commonly confused - are dealt with in the scientific literature. The study shows that, although there is a diversity of aspects that comprise the complex concepts of presence, flow, and narrative absorption, some themes are more frequently related to these concepts than others.

 This work is especially important in a context in which new VR tools are being increasingly used. Perhaps, in the future, we will focus less on trying to find a consensus towards the meaning of "flow" or "presence" and will focus on more pragmatic aspects such as "losing track of time"?

 One aspect I particularly like about this article is that all conclusions are based on data, and I could not find any overstatement regarding the study. It is clearly written. Used data is available for replication. The discussion session is especially exciting.

 Some minor observations:

1. In the Rationale (if there is space): the text states: "Overlapping concepts have been formulated in different fields according to specific disciplinary interests and based on knowledge within each field". This is absolutely true, and it is the main problem tackled in this work. Could the author provide one or two examples of these different definitions?
2. In the third paragraph of the discussion, change "and the comprehension of the content of the story" to "the understanding of the story's narrative and characters".
3. The idea of "reality non mediated by technology", which appears in the synthesis of results, refers to "actual, physical reality", as opposed to its virtual counterpart. Is this correct? Could the author include one or two sentences explaining the difference between mediated and non-mediated reality, and why is this different from differentiating "virtual" from "real"? Or, at least, provide a reference for further reading?

None of these notes take away the relevance and quality of this work. I congratulate the author and have no further comments on this article.

Are sufficient details of the methods and analysis provided to allow replication by others?

Yes

Are the rationale for, and objectives of, the Systematic Review clearly stated?

Yes

Is the statistical analysis and its interpretation appropriate?

Yes

Are the conclusions drawn adequately supported by the results presented in the review?

Yes

Reviewer Expertise:

I have worked with presence and flow, although I currently do not use these terms anymore (due to the exact reasons stated in this article). Currently, I work with technology-mediated musical interactions and this article is highly interesting to my research.

I confirm that I have read this submission and believe that I have an appropriate level of expertise to confirm that it is of an acceptable scientific standard.

# Response

**Published:** 2021-07-15

**Authors:** Federico Pianzola

**Affiliations:** University of Milan Bicocca, Italy

Thank you very much for your positive feedback. Regarding your comments:

1. I will add examples of how different definitions of the same concept are based on specific disciplinary knowledge.
2. I agree that understanding characters is often considered an important aspect of subjective-phenomenal states related to narrative.
3. I will elaborate on the difference between mediated and non-mediated experiences, a topic which I treated more at lengths in a complementary article based on this scoping review: Pianzola, F., Riva, G., Kukkonen, K., & Mantovani, F. (2021). Presence, flow, and narrative absorption: An interdisciplinary theoretical exploration with a new spatiotemporal integrated model based on predictive processing. *Open Research Europe*, *1*(28), 1–25. https://doi.org/10.12688/openreseurope.13193.1
